# The Use of Human Adipose-Derived Stem Cells in the Treatment of Physiological and Pathological Vulvar Dystrophies

**DOI:** 10.1155/2016/2561461

**Published:** 2016-01-10

**Authors:** Maria Giuseppina Onesti, Sara Carella, Simona Ceccarelli, Cinzia Marchese, Nicolò Scuderi

**Affiliations:** ^1^Department of Surgery “P. Valdoni”, Sapienza University of Rome, Via Giovanni Maria Lancisi 2, 00161 Rome, Italy; ^2^Department of Experimental Medicine, Sapienza University of Rome, Italy

## Abstract

“Vulvar dystrophy” is characterized by chronic alterations of vulvar trophism, occurring in both physiological (menopause) and pathological (lichen sclerosus, vulvar graft-versus-host disease) conditions. Associated symptoms are itching, burning, dyspareunia and vaginal dryness. Current treatments often do not imply a complete remission of symptoms. Adipose-Derived Stem Cells (ADSCs) injection represents a valid alternative therapy to enhance trophism and tone of dystrophic tissues. We evaluated efficacy of ADSCs-based therapy in the dystrophic areas. From February to April 2013 we enrolled 8 patients with vulvar dystrophy. A biopsy specimen was performed before and after treatment. Digital photographs were taken at baseline and during the follow-up. Pain was detected with Visual Analogue Scale and sexual function was evaluated with Female Sexual Function Index. All patients received 2 treatments in 3 months. Follow-up was at 1 week , 1 and 3 months, and 1 and 2 years. We obtained a significant vulvar trophism enhancement in all patients, who reported pain reduction and sexual function improvement. Objective exam with speculum was easy to perform after treatment. We believe ADSCs-based therapy finds its application in the treatment of vulvar dystrophies, since ADSCs could induce increased vascularization due to their angiogenic properties and tissue trophism improvement thanks to their eutrophic effect.

## 1. Introduction

Vulvar dystrophy is a condition which may occur in women both in physiological and in pathological conditions. Associated symptoms may include itching, burning, dyspareunia, vaginal dryness and bleeding. Vaginal dystrophy commonly affects postmenopausal women, with prevalence ranging from 10% to 50% [1, 2] and it is estimated that up to 45% of all women are symptomatic [3]. The aetiology of vulvovaginal atrophy among postmenopausal women is most commonly explained by the decrease in circulating estrogen associated with the menopausal transition, which has an adverse effect on skin collagen and elasticity [4]. Even while taking systemic estrogen, 10% to 20% of women may still have residual symptoms [5]. Symptoms related to estrogen reduction may also occur during other stages of women's lives in response to events associated with sustained decreases in estrogen such as lactation or also related to chemotherapy that induces premature ovarian failure in 14–100% of cases. These patients are at high risk of transient or permanent amenorrhea, and, for those women who continue to menstruate or who recover their cycles, there is an additional long-term risk of premature ovarian failure. In particular, breast cancer treatment increases the prevalence of vulvar dystrophy because the surgical, endocrine, and chemotherapeutic agents used in its treatment can cause or exacerbate this condition. An iatrogenic cause of vulvar dystrophy is related to allogeneic hematopoietic stem cell transplant. This procedure has found a place in the treatment of a variety of malignant and nonmalignant diseases of the bone marrow and immune system. However, it may be complicated by chronic graft-versus-host disease (GVHD), due to activation of donor immunological cells against host tissues. GVHD may occur in acute or chronic forms. The skin, mouth, eyes, liver, and intestines are the organs most commonly involved in chronic GVHD. Genital tract involvement can occur in nearly 25% of patients with GVHD. Vulvar scarring can lead to vaginal stenosis and introital reduction and may also result in labial fusion [6]. These manifestations can lead to some complications as hematometra, hematocolpos, and abscesses. Hypoestrogenism from chemotherapy-induced premature ovarian failure and chronic GVHD can both cause vulvar or vaginal pain and irritation. Lichen sclerosus (LS) is a chronic immune-mediated inflammatory skin disorder of poorly understood aetiology [7, 8], which may be localized anywhere on the body but has a predilection for the anogenital area [9]. Although disease onset has been reported at all ages, it occurs most commonly in women in their fifth or sixth decade of life. Skin changes of the vulva initially begin with white, polygonal papules that coalesce into plaques, with skin atrophy and perifocal erythema. Additionally, subcutaneous bleeding with ecchymosis or hematosis and fissures with superficial ulceration and erosion may occur. Late progressive symptoms include thinning of the mucosa, edema and fibrosis of the dermis, shrinkage of the labia, and agglutination of the labia minora that can lead to stenosis of the introitus. Moreover, while the disorder is considered benign, some women with vulvar LS may later develop squamous cell carcinoma (SCC) of the vulva and some women have concomitant SCC when initially diagnosed with LS [10]. Vulvar dystrophy may negatively affect the entire sexual response cycle, inducing significant changes in desire, arousal, orgasm, and satisfaction at menopause and beyond. Patients may be embarrassed by the disfiguring changes that may occur and avoid sexual intimacy. Furthermore these patients may have increased risk to develop bacterial vaginosis due to vaginal pH changes and urinary tract infection or stress urinary incontinence. Main treatments to solve urogenital and sexual dysfunction due to vulvovaginal tissue alteration currently include drug therapy and lubricants, but in the majority of patients these therapies do not imply a complete remission of symptoms. Recent studies emphasized that adipose tissue is a rich source of adult stem cells, the so-called Adipose-Derived Stem Cells (ADSCs) [11–13]. We tested the hypothesis that the vulvar application of ADSCs can enhance the trophism and tone of dystrophic tissues.

## 2. Materials and Methods

### 2.1. Patients

The clinical trial protocol, conformed to the guidelines of the Declaration of Helsinki (1964), was approved by the Ethical Committee of our institution (Ref.1834/25.03.10). From February to April 2013 we enrolled 8 patients affected with vulvar dystrophy of average age of 56.5 (between 38 and 75 years old). Among them, one patient was in postmenopause, 2 women were affected with vulvar GVHD, and 5 were affected with LS. At each visit, personal, anamnestic, and objective data were collected. Our study was conducted with the patients' understanding and consent. Patients were informed of the purpose and objective of the investigation and were asked for their written informed consent for participation and publication of the data obtained during the course of the study. Inclusion criteria called for vulvar dystrophy's symptoms and signs, patients' refusal of lipofilling, and patients nonresponders to traditional therapy. Exclusion criteria were pregnancy or lactation and associated pathologies contraindicating the proposed treatment.

All patients received 2 treatments in 3 months. Follow-up was at 1 week and 1 and 3 months and at 1 and 2 years.

### 2.2. Surgical Techniques

The adipose tissue collection was performed through liposuction from the abdominal region or trochanteric areas, depending on the easier access to a sufficient amount of adipose tissue. The sampling was carried out under local anesthesia, using a modified Klein solution (1 liter of sodium chloride 0.9%, 20 mL of lidocaine 2%, and 1 mL of epinephrine 1 : 200,000) through a single hole of 3 mm blunt cannula connected to a 10 cc Luer-lock syringe. The harvested fat was centrifuged at 1500 rpm for 2 minutes. After centrifugation, the oil layer (upper level) and the aqueous layer (lower level) were eliminated from the syringe. The middle layer containing adipocytes and stromal vascular component was sent to the laboratory for cell cultivation within 1 hour.

### 2.3. Cell Isolation and Culture

The fat was transferred into a sterile tube and washed extensively with sterile phosphate-buffered saline (PBS) containing 2% PSG to remove contaminating debris and red blood cells. The stromal vascular fraction (SVF), containing the ADSCs, was then pelleted by centrifugation for 5 min at 2000 rpm [14] and then resuspended in DMEM-Ham's F-12 medium (vol/vol, 1 : 1) (DMEM/F-12, Gibco) supplemented with 20% FBS, 100 U/mL penicillin, 100 *μ*g/mL streptomycin, and 2 mM L-glutamine and plated in a 75 cm^2^ tissue culture flask coated with collagen type IV.

ADSCs were self-selected out of the SVF during subsequent tissue culture passages, on the basis of their ability to adhere to the plastic tissue cultureware, and maintained in a 5% CO_2_ incubator at 37°C in a humidified atmosphere, with medium change twice a week. Cell viability was assessed by using the trypan blue exclusion assay. A homogeneous population of ADSCs was subsequently checked by determining growth kinetics and by analyzing the surface-marker expression profile.

### 2.4. Preparation of Cell-Enriched Hyaluronic Acid

Primary cultures of ADSCs derived from each patient were expanded in order to obtain an adequate number of 75 cm^2^ flasks at 95–100% confluence. Once the appropriate number of cells was reached for each patient, cells were detached with 0.5 mM EDTA/0.05% trypsin for 5 min at 37°C and counted. Then, ADSCs were centrifuged at 1500 rpm for 10 minutes, washed twice in phosphate-buffered saline to remove serum, and resuspended in an adequate volume of synthetic stabilized low molecular weight hyaluronic acid solution (a 1.6% solution of synthetic HA, without chemical modifications and with a molecular weight of 1 × 10^3^ KDa, very similar to the endogenous HA) at the standard concentration of 5 × 10^5^ cells/mL, to vehicle stem cells and to facilitate subsequent infiltration. After mixing, the suspension was kept under ambient conditions for 10 to 15 minutes to allow cell adherence to the hyaluronan matrix. Homogeneous dispersion of the cells within the gel was ensured by microscopical observation. Then, the cell-supplemented hyaluronic acid solution was loaded into an injection syringe and carried to the operating room.

### 2.5. ADSCs Infiltration

Usually after 3 weeks, the patient went back to the operating room for the inoculation of autologous ADSCs into the dystrophic areas. The procedure was performed in local anesthesia with sedation, with the patient in the dorsal lithotomy. The infiltration was done using two 2 mL syringes provided with a 30-gauge 1/2 needle by means of multiple passes in the subcutaneous plane of labia minora. We employed 2 mL of hyaluronic acid for each patient, keeping a constant rate of 5 × 10^5^ cells for each mL of hyaluronic acid. All patients were dismissed the following day.

### 2.6. Clinical Evaluation

The evaluated parameters were tissue trophism, pain, and sexual satisfaction. Tissue trophism was evaluated by photographic documentation and biopsy performed before and after treatment. Photographs were taken in occasion of follow-up utilizing the same parameters (same camera Canon EOS 500D, same shooting distance of 50 cm, and same anatomical landmarks). Pain was estimated at T0 and weekly according to the Visual Analogue Scale (VAS). A range from 1 (no pain) to 10 (maximal sensation of pain) was considered. Sexual function was assessed by the Female Sexual Function Index (FSFI), performed before and after treatment. It consists of 19 questions on the sexual activity performed in the last four weeks. It enables assessment of six sexual functioning domains: desire, arousal, lubrication, orgasm, satisfaction, and discomfort/pain [15]. Subdomains are scored considering the values of each question and its respective conversion factors, and total FSFI Score was calculated as the sum of the six results, ranging from 2 to 36. Better levels of sexual function were indicated by highest scores.

## 3. Results

All patients were followed postoperatively at least for 2 years, with periodical medical assessments at 1 week, 1 and 3 months, and 1 and 2 years.

**Figure 1:**
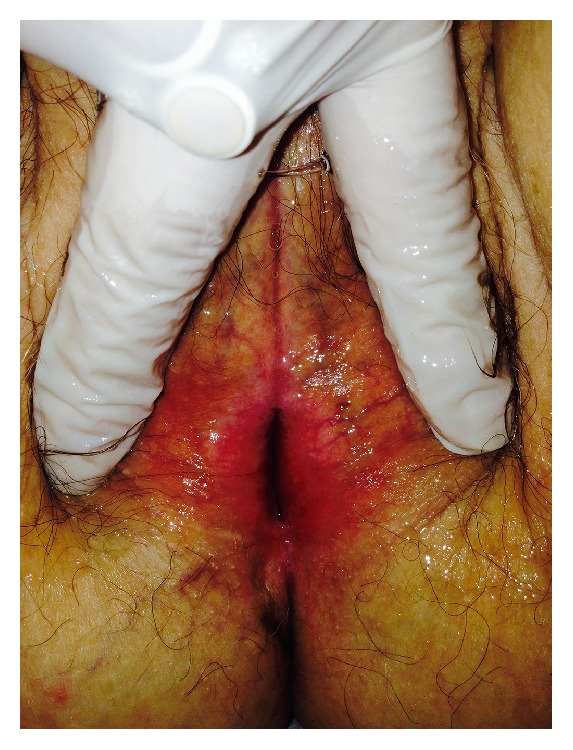
Before ADSCs.

**Figure 2:**
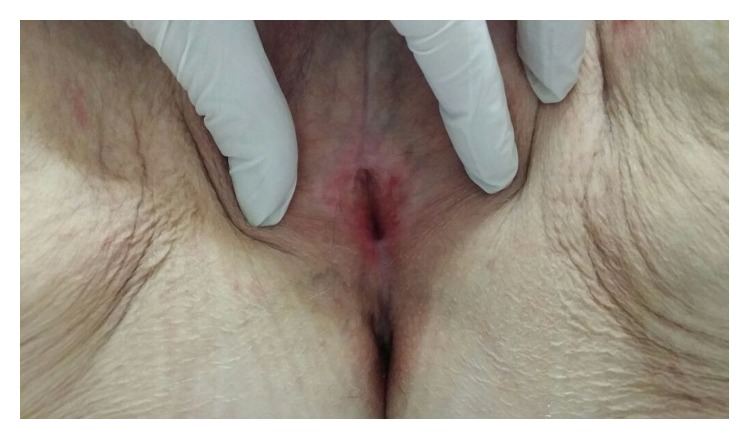
After ADSCs.

**Figure 3:**
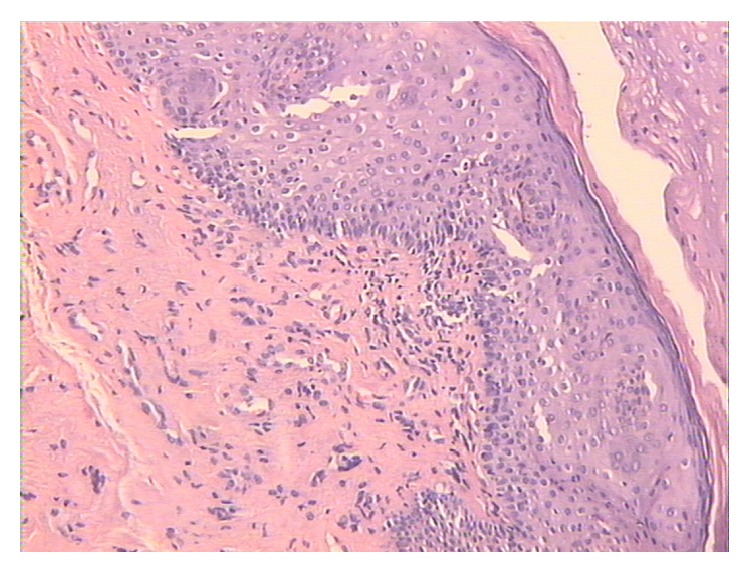
Hematoxylin-eosin staining ×10. Epidermis with mild parakeratotic hyperkeratosis ectatic capillaries, sclerotic dermis, and inflammatory infiltrate.

**Figure 4:**
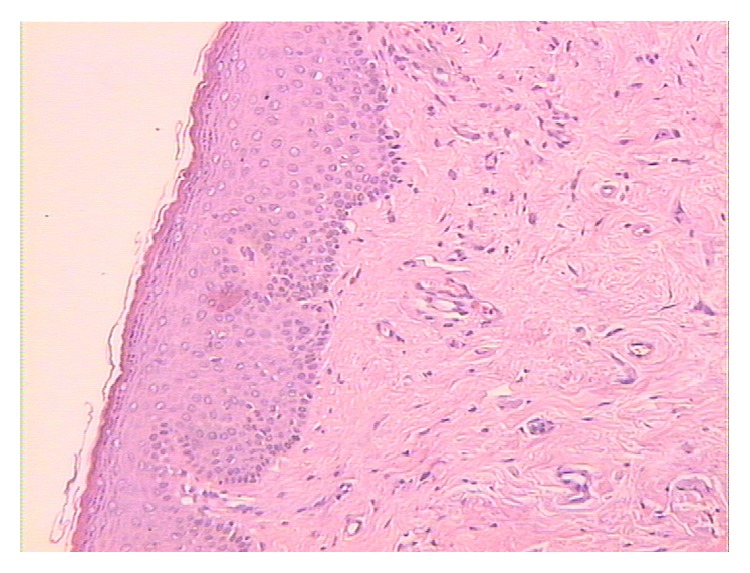
Hematoxylin-eosin staining ×10. Reduction of dermis sclerosis, capillaries less dilatated, inflammatory infiltrate dramatically reduced.

Just one month after the first ADSCs infiltration, improvement of vulvar trophism was clinically observed, enhancing progressively over time. Pain reduction was already documented at one month after the first treatment; dramatic pain reduction was obtained after 1 year. Sexual function improved significantly at one month after the second treatment. Overall results in terms of pain reduction, vulvar trophism, and sexual function improvement were well-maintained at 2 years' follow-up. In particular, photographic documentation at 2 years' follow-up revealed an improvement of vulvar trophism, showing a pink vulvar skin color, a restoration of elasticity, and the disappearing of erythematous areas. We observed that speculum exam was easier to perform and painless as indicated from the patients. A loss of vaginal rugae, vaginal pallor, and petechiae was evident in the patient affected in menopause. Histological exam in menopause patient demonstrated improvement of the elastic network. In patients with LS, showing extensive erythema of the labia minora extending onto the interlabial sulci (which also show lichenification) and the vaginal introitus before treatment (Figure 1), we observed that erythema was dramatically reduced and disappeared at level of the interlabial sulci and the vaginal introitus after ADSCs infiltration (Figure 2). In patients affected with GVHD, histological examination documented apoptotic cells in the basal layer, epithelial detachment from the thinning mucosa, collagen hyalinosis, and monocytic infiltrate in the submucosa. After treatment with ADSCs, a regression of the morphological alterations of epithelial cells and full attenuation of inflammatory signs in the connectival tissue were observed. Biopsy specimens of patients affected with LS displayed epithelial acanthosis, appendageal hyperkeratosis and dermal modifications with hyalinization, lymphocytic infiltrate with a longitudinal band in the medium dermis, wide ectatic capillaries, and lymphocyte tagging along the basement membrane. After treatment with ADSCs, the dermis sclerosis was significantly reduced, capillaries that resulted were less dilated, and inflammatory infiltrate was dramatically reduced (Figures 3 and 4). According to the VAS, a remarkable pain reduction could be observed in all patients, as shown in Table 1. Moreover, FSFI revealed that all patients obtained an improvement of sexual function after ADSCs treatment, as documented in Table 2. Our method was efficacious regardless of the aetiology of vulvar dystrophy.

## 4. Discussion

Vulvar dystrophy negatively impacts women's lives, but women lack knowledge of the subject and are hesitant to consult healthcare professionals, who should proactively initiate discussions regarding appropriate treatment options. Main treatments to solve urogenital and sexual dysfunction due to vulvovaginal tissue dystrophy currently include drug therapies and lubricants but in the majority of patients they do not imply a complete remission of symptoms. Lubricants can be used to decrease immediate irritation during coital activity, but there is no evidence that these products have any long-term therapeutic effect. Systemic hormone replacement is indicated for women who are seeking to treat a variety of symptoms associated with the estrogen deprivation of menopause, such as hot flushes and sleep disturbance, but, in some women, it is not an effective method for relieving vulvovaginal atrophy [16]. Furthermore, estrogens cannot be used in women affected with breast cancer undergoing the hormone suppressive therapy. To date, new therapeutic approaches have been experimented. Hyaluronic acid (HA) infiltration is recently used for vaginal rejuvenation. HA is an unbranched, nonsulphated polysaccharide, and it is biocompatible, nontoxic, and biodegradable. Due to its high water-binding capacity or hygroscopicity, it induces tissue hydration and lubrication [17]. In the skin, HA is produced intracellularly at the cell membrane of fibroblasts by HA-synthases [18] and extruded directly into the extracellular matrix. Since HA is chemically and structurally identical, regardless of its origin, allergic reactions are rare and skin tests before injection are not necessary. This led to the use of HA in several tissue engineering areas, such as aesthetic surgery. Moreover, previous observations suggested that nonanimal stabilized hyaluronic acid may confer filling effects, attributable to its stimulatory effect on collagen production [19], but a major drawback of HA is the lack of a tissue regeneration, with the filling material that is only degraded and not replaced by autologous tissue. Therefore, it is almost completely reabsorbed after 1 month, and its effects are only temporary. In the last few years, autologous fat transplantation has become the first-choice treatment to restore volumes and to achieve structural modifications. This approach, using the patient's own body fat as a natural filler, takes advantage of its abundance and accessibility, thus avoiding complications associated with foreign materials [20]. Nevertheless, many studies demonstrated that the most part of adipose tissue grafts is reabsorbed through time or replaced by fibrous tissue, and they also display a low rate of survey due to partial necrosis [21]. Lipofilling benefits have already been proved in dystrophies and vulvar rejuvenating treatments. As outlined above, the adipose tissue is a rich source of ADSCs. This stem cell reservoir can be easily obtained from a very small amount of liposuction aspirate (1–5 cc) or from adipose tissue biopsy. Furthermore, ADSCs possess the ability to differentiate into various cell types, including adipocytes, chondrocytes, osteoblasts, and myoblasts. Therefore, they may represent a promising approach to cell-based therapies [11–13]. Besides, it has been recently demonstrated that ADSCs show angiogenic properties and could also exert some immunomodulatory activities, including a suppressive response on collagen-reactive T cells and the capacity to restore immune tolerance by inhibiting the inflammatory response* in vivo* [13]. ADSCs infiltration finds its application in the treatment of physiological and pathological vulvovaginal dystrophies, since these cells could induce increased vascularization, due to their angiogenic properties, and an improvement of tissue trophism, due to their eutrophying effect. In particular, in patients affected with pathological inflammatory dystrophies, a reduction of the typical pain and burning sensation could be obtained thanks to the immunomodulatory properties of the ADSCs, which may act by reducing inflammation that represents the algogenic stimulus of these pathologies. Therefore, it is possible to obtain the appropriate number of cells to treat different pathologies, by means of conveyance with a low molecular weight hyaluronic acid gel, thus allowing an easy and nontraumatic infiltration in the affected areas. Nonanimal stabilized hyaluronic acid represents an ideal scaffold that can provide structural support and a favorable environment for growing cells, and it is a natural component of commonly used injectable soft-tissue filler [22]. Moreover, a further advantage of this procedure is the reduction of operating time and the greater compliance of patients and surgeons in comparison with the traditional invasive techniques.

## 5. Conclusion

ADSCs injection represents the evolution of a standardized therapy, such as conventional lipofilling, and a valid alternative method to the injection of hyaluronic acid alone for biorevitalizing purposes.

On the basis of our experience we advocate that ADSCs infiltration in dystrophic vulvar areas is a safe method, easier to be performed with respect to the traditional technique, noninvasive, and requiring short time. It could represent a valid therapeutic option when patients refuse or present conditions contraindicating an invasive procedure. In agreement with the positive results previously achieved with this technique in pathologies characterized by tissue scleroatrophy such as scleroderma [14], we obtained encouraging results in patients suffering from vulvar dystrophies, even in the long-term, in terms of cutaneous, subcutaneous, and mucous trophism improvement. Our patients experienced a restoration of the anatomy, altered by the pathology, and could improve their quality of life. ADSCs treatment leads to the reacquisition of vaginal and periurethral tonicity, thus solving problems related to the hypotrophic involutive processes affecting the genitourinary tract. After treatment our patients performed objective exam, Pap test, and other gynaecological procedures, without pain.

## Figures and Tables

**Table 1:** Visual Analogue Scale (VAS) score before and after treatment.

Cases	Patients (*N*)	VAS score	
		Before ADSCs	After ADSCs
Menopause	1	7	1

GVHD	1	9	2
	1	8	1

LS	1	8	1
	1	7	1
	1	8	1
	1	9	2
	1	8	1

**Table 2:** Female Sexual Function Index (FSFI) before and after treatment.

Cases	Patients (*N*)	FSFI	
		Before ADSCs	After ADSCs
Menopause	1	10	30

GVHD	1	15	30
	1	20	30

LS	1	15	36
	1	14	35
	1	12	35
	1	10	36
	1	10	36
